# An efficient cardiac mapping strategy for radiofrequency catheter ablation with active learning

**DOI:** 10.1007/s11548-017-1587-4

**Published:** 2017-05-05

**Authors:** Yingjing Feng, Ziyan Guo, Ziyang Dong, Xiao-Yun Zhou, Ka-Wai Kwok, Sabine Ernst, Su-Lin Lee

**Affiliations:** 1 Hamlyn Centre and Department of Computing, Imperial College London, London, UK; 2 Department of Mechanical Engineering, The University of Hong Kong, Hong Kong, China; 3 Royal Brompton Hospital, London, UK

**Keywords:** Radiofrequency catheter ablation, Cardiac mapping, Learning from demonstration, Active learning, Catheter robot guidance

## Abstract

**Objective:**

A major challenge in radiofrequency catheter ablation procedures is the voltage and activation mapping of the endocardium, given a limited mapping time. By learning from expert interventional electrophysiologists (operators), while also making use of an active-learning framework, guidance on performing cardiac voltage mapping can be provided to novice operators or even directly to catheter robots.

**Methods:**

A learning from demonstration (LfD) framework, based upon previous cardiac mapping procedures performed by an expert operator, in conjunction with Gaussian process (GP) model-based active learning, was developed to efficiently perform voltage mapping over right ventricles (RV). The GP model was used to output the next best mapping point, while getting updated towards the underlying voltage data pattern as more mapping points are taken. A regularized particle filter was used to keep track of the kernel hyperparameter used by GP. The travel cost of the catheter tip was incorporated to produce time-efficient mapping sequences.

**Results:**

The proposed strategy was validated on a simulated 2D grid mapping task, with leave-one-out experiments on 25 retrospective datasets, in an RV phantom using the Stereotaxis Niobe^®^ remote magnetic navigation system, and on a tele-operated catheter robot. In comparison with an existing geometry-based method, regression error was reduced and was minimized at a faster rate over retrospective procedure data.

**Conclusion:**

A new method of catheter mapping guidance has been proposed based on LfD and active learning. The proposed method provides real-time guidance for the procedure, as well as a live evaluation of mapping sufficiency.

## Introduction

Radiofrequency catheter ablation (RFCA) is a minimally invasive procedure for the treatment of cardiac arrhythmias. An ablation catheter is advanced from the groin region to the heart to be used to create a line of lesions on the endocardium, in an effort to block the trigger points of the arrhythmia and its propagation. In the *mapping* stage of the procedure, an operator collects position and electroanatomical information at a series of points along the endocardium, using a catheter. This information is then used to create electrical activation and voltage maps using an electroanatomical mapping system (EAMS) such as the CARTO^®^ system. Then in the ablation stage, the operator delivers RF energy via the catheter tip in order to ablate the trigger points of the arrhythmia.

The mapping stage is critical for the identification of the cardiac pathology, as well as the location of trigger points. A precise map from a large sample of mapping points is preferable but a prolonged mapping time might result in the induced tachycardias terminating before the trigger points are successfully identified and also pose a danger to patients. In patients with congenital heart disease, such as Tetralogy of Fallot (ToF), identifying the trigger points can be difficult due to the complex anatomy. Robotic catheter platforms, such as the Stereotaxis system, have enabled access to regions previously inaccessible by manual catheters but do not reduce procedure time. An efficient mapping strategy is therefore necessary.

In this work, we address the difficulty in developing a mapping strategy for cardiac voltage mapping for congenital ToF RVs. The goal is to efficiently identify the low-voltage areas representing the scar-related tissues; therefore, two requirements should be fulfilled: (i) to widely spread the mapping points so that the anatomy is covered as much as possible and (ii) to focus on areas with high gradients on electrical values as they are possibly the borders of scar tissues. Previous work for the same clinical application included a geometry-based method [15] which reduced the mesh of a heart chamber to a small number of vertices which were computed to have a maximal coverage of anatomy and then arranged the vertex order to form the mapping sequence. However, this work only addressed requirement (i), and the quality of the resulted electrical map was not evaluated.

Here, we present a surrogate model-based active-learning approach to automatically generate a mapping sequence during the mapping procedure, using Gaussian process (GP) regression for estimating the cardiac voltage map. The prior knowledge for GP is learned from the demonstrated mapping sequences of an expert operator over previous voltage mapping procedures for congenital ToF RVs. The mapping process reduces the uncertainty greedily to mimic the decision process of the operator, while updating the model with the observations to best fit the electrical pattern of the current RV. Under the uncertainty-based sampling, the whole anatomy is explored as much as possible, fulfilling requirement (i). The sampling points selected based on the electrical pattern of the current RV also encode requirement (ii). Experiments were performed on simulated data, retrospective in silico patient data, and on phantom data with robotic catheters with results of the proposed method compared to the original expert paths and an existing geometry-based method.

## Background

### Active learning

In cardiac mapping, the operator chooses a sequence of mapping points on the surface of the endocardium to maximize the knowledge about the target region; this can be seen as a sampling problem to maximize the information gain. At the same time, steering the mapping catheter to an intended position has an associated travel cost. Thus, the problem is formulated as taking only a small number of mapping points from a large number of candidates to construct a cardiac electrical map. This lies at the heart of the active learning problem, which tries to extract maximum information of an unknown function by issuing as few queries as possible to the function.

**Fig. 1:**
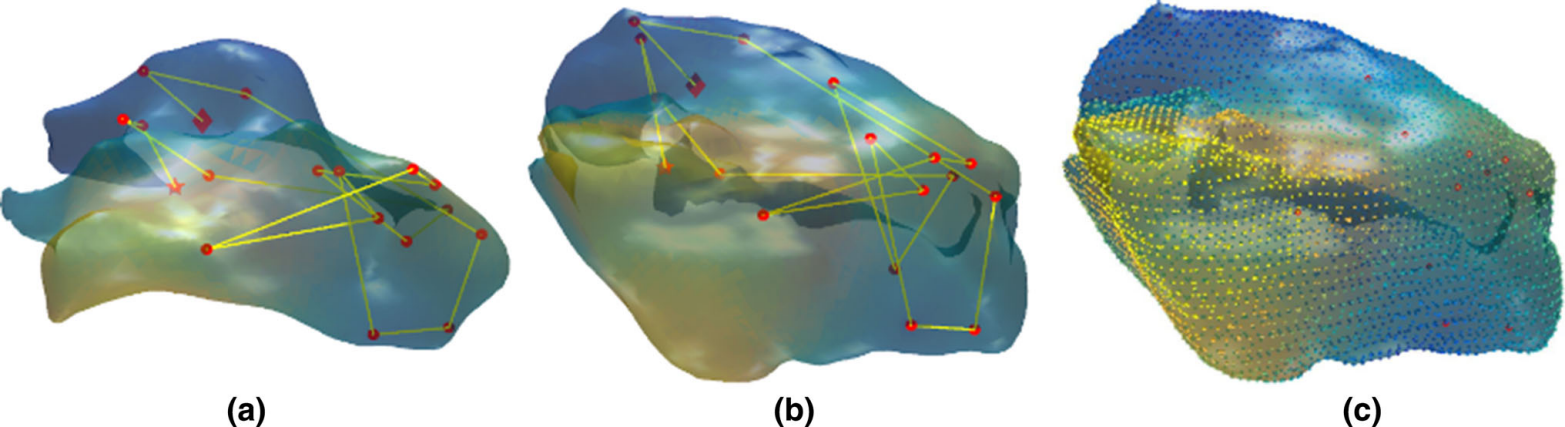
The mapping points (*red dots*) taken in dataset 2 with their order shown by the *yellow line*. The *surface colours* represent the voltage values estimated by the CARTO^®^ system, and the *vertex colours* in (**c**) show the estimated voltage values by GP regression based on the mapping points taken. The mapping sequence and voltage values of the intraoperative mesh in (**a**) were both projected to the preoperative mesh in (**b**) using a nearest neighbour search. In (**c**), the estimated voltage values (*vertex colours*) are similar to the voltage values exported from the CARTO^®^ system (*surface colours*)

A surrogate model is used by an agent to simulate the world upon which it acts and in which it receives feedback. In the surrogate model-based active learning, the agent has a model representing its knowledge about the world. It has two goals to optimize synchronously, in order to reap maximum reward (knowledge) from the real world: *exploration* to increase the accuracy of the model and *exploitation* to pick the best action based on the model.

In [13], an active-learning-like approach was performed to plan the activation mapping process in RFCA. With a 4D vector to represent the cardiac electrical conduction direction, the optimal path was computed by greedily selecting the measurements as the ones that would maximally reduce the posterior uncertainty of the Bayesian model. However, our mapping target, i.e. low-voltage scar tissue, cannot be faithfully represented using a 4D vector.

### Gaussian process

GP describes data as a collection of random variables where any *k* random variables have a *k*-variate joint Gaussian distribution [11]. Let ${\mathbf {X}}$ be the input of observed points, ${\mathbf {f}}$ be the training output of ${\mathbf {X}}$, and ${\mathbf {y}} = {\mathbf {f}} + \varepsilon$ be the test output (i.e. the observed value), where $\varepsilon$ represents the white noise of the observation which is assumed to have a Gaussian distribution $\varepsilon \sim N(0, \sigma ^2 I)$. Let ${\mathbf {X}}_*$ be a set of query points, with their predicted values represented in ${\mathbf {f}}_*$. Assuming the mean is zero, the covariance function can be calculated with a kernel function $K(\cdot , \cdot )$, and thereby, we get

$$\begin{aligned} \begin{bmatrix} {\mathbf {y}} \\ {\mathbf {f}}_*\end{bmatrix} \sim N \begin{pmatrix} 0, \begin{bmatrix} K({\mathbf {X}}, {\mathbf {X}}) + \sigma ^2 I \ \ \ K({\mathbf {X}}, \mathbf {X_*}) \\ \ \ K(\mathbf {X_*}, {\mathbf {X}}) \ \ \ \ \ \ \ \ K(\mathbf {X_*}, \mathbf {X_*}) \end{bmatrix} \end{pmatrix} \end{aligned}$$

The posterior probability is the conditional normal distribution as follows.

1 $$\begin{aligned}&{\mathbf {f}}_*|{\mathbf {X}}_*, {\mathbf {y}}, {\mathbf {X}} \sim {N}(\mu _*, \varSigma _*), \ \text {where} \nonumber \\&\mu _*= K({\mathbf {X}}_*, {\mathbf {X}})[K({\mathbf {X}},{\mathbf {X}}) + \sigma ^2 {\mathbf {I}}]^{-1}{\mathbf {y}} \nonumber \\&\varSigma _*= K({\mathbf {X}}_*, {\mathbf {X}}_*) - K({\mathbf {X}}_*,{\mathbf {X}})[K({\mathbf {X}}, {\mathbf {X}}) {+} \sigma ^2 {\mathbf {I}}]^{-1} K({\mathbf {X}}, {\mathbf {X}}_*)\nonumber \\ \end{aligned}$$

A GP model was adopted as the surrogate model for active learning in this work for two reasons: firstly, GP posterior covariance conditioned on a set of observed points can reflect the uncertainty of the unmapped sites (shown in Eq. 1); and secondly, GP has a good regression performance, as it can approximate a smooth function flexibly by choosing different kernels, and the regression prevents over-fitting. As a surrogate model, GP regression was adopted to estimate the voltage map.

## Methods

### CARTO^®^ data export and preprocessing

In cardiac mapping, the preoperative meshes of the patient heart anatomy are created either by using computed tomography (CT) or magnetic resonance imaging (MRI) scans. During the procedure, by using the trajectory of the mapping catheter, a mesh is created by an EAMS to approximate the geometry of the targeted region of the heart in real time. This mesh is also overlaid with a colour layer which represents map quantities such as voltages or activation time [2], which is calculated by the EAMS based on the mapping points. We refer to this mesh created during the procedure as an intraoperative mesh. During the procedure, the intraoperative mesh was manually registered with the preoperative mesh by a technician to help with tracking the catheter.

Retrospective mapping data from 25 cardiac ablation procedures performed on RVs of congenital ToF patients was exported from the CARTO^®^ 3 navigation system (Biosense-Webster, Diamond Bar, CA, USA) at the Royal Brompton Hospital, London, UK. All cases were performed by a single expert operator. Mapping data (3D mapping point positions, preoperative meshes from MRI, and intraoperative meshes created by the CARTO^®^ system) of the RVs were used in this study.

Duplicate mapping points were removed to ensure the stability of GP regression. The voltage values on the intraoperative mesh and all the mapping points (Fig. 1a) were projected onto the triangulated-mesh surface of the preoperative mesh by nearest neighbour search (Fig. 1b). In Fig. 1c, the voltage values estimated by GP regression from mapping points are closely comparable to the exported voltage values. Due to the similarity, GP regression was used to estimate the voltage values in this work.

### Kernel learning for GP from expert mapping sequences

In favour of using prior knowledge from medical studies to select the kernel form (as in [7]), a variety of different kernels were used to create the estimated voltage map, and the one most accurately representing the data was selected.

For the kernel training, the 3D positions of *N* mapping points were contained in the $N \times 3$ vector ${\mathbf {X}}_{\mathrm{train}}$ as training input and the corresponding voltage values are represented by the $N \times 1$ vector ${\mathbf {y}}_{\mathrm{train}}$ as training output (note that this training output differs from the one defined in the “Gaussian process” section). For each of the 24 out of 25 total datasets, ${\mathbf {X}}_\mathrm{train}$ and ${\mathbf {y}}_\mathrm{train}$ were concatenated without any additional modification. The concatenation still preserves the one-to-one relationship between the 3D position and the voltage value of each mapping point. The remaining dataset was used to test the mapping strategy which will be described in the “Implicit exploration (IE) approach” section.

In conjunction with the gradient-descent method [11], linear, square exponential, Matérn, and rational quadratic kernels were used as the potential kernel forms with randomly initialized hyperparameter values. As the Matérn kernel produced the least mean square root error with the true voltage, the resultant hyperparameter of the Matérn kernel was used as the initial hyperparameter value $\theta _0$ for the later step. The kernel learning step can be seen as compiling a history of cases for a particular cohort of patients (congenital ToF RVs) as prior knowledge. In the online planning step (to be described), this prior knowledge will be updated as more observations are taken.

### Implicit exploration (IE) approach

From information theory, a set of random variables is the optimal set for sampling when it has the maximum information entropy. Directly finding such a set for a given cardinality has been proven to be NP-hard [5], but the heuristics of picking the most uncertain point $\tilde{{\mathbf {x}}}$ sequentially to form the set can still achieve $(1- 1/\varepsilon )$ optimality [9]. Let ${\mathbf {V}}$ represent the total set of points to estimate and ${\mathbf {A}}$ a set of observations. Let $\bar{{\mathbf {A}}} = {\mathbf {V}} - {\mathbf {A}}$, then the most uncertain point from $\bar{{\mathbf {A}}}$ is

$$\begin{aligned} \tilde{{\mathbf {x}}} = \mathop {{{\mathrm{arg\,max}}}}\limits _{{\mathbf {x}}' \in \bar{{\mathbf {A}}}} {\mathbb {H}}({\mathbf {f}}'| {\mathbf {x}}', {\mathbf {A}}) \end{aligned}$$

where ${\mathbb {H}}({\mathbf {f}}'| {\mathbf {x}}', {\mathbf {A}})$ can be calculated based on a normal distribution form

$$\begin{aligned} {\mathbb {H}}[ {\mathbf {f}}'| {\mathbf {x}}', {\mathbf {A}}] = \frac{1}{2} \log |\varSigma '| + \frac{D}{2}(\log 2 \pi e) \end{aligned}$$

where $\varSigma ' = \varSigma _*$ from Eq. (1). With a fixed kernel form, the computation of the posterior entropy of the unobserved points depends solely upon the kernel hyperparameter $\theta$. Therefore, we introduce the notation of ${\mathbb {H}}[{\mathbf {f}}'| {\mathbf {x}}', {\mathbf {A}}, \theta ]$ for the entropy estimation for the GP model based on a Matérn kernel form with hyperparameter $\theta$.

If there is an accurate kernel hyperparameter $\theta = \theta _0$ to reflect the ground truth, the agent only needs to consider exploitation for the estimation of the ground truth. This approach, as shown in Eq. (2), is denoted as pure exploitation (PE).

2 $$\begin{aligned} {\tilde{\mathbf {x}}}_\mathrm{PE} = \mathop {{{\mathrm{arg\,max}}}}\limits _{{\mathbf {x}}' \in \bar{{\mathbf {A}}}} {\mathbb {H}}[\mathbf {f'} | {\mathbf {x}}', {\mathbf {A}}, \theta _0] \end{aligned}$$

PE iteratively selects the next mapping point ${\tilde{\mathbf {x}}}_\mathrm{PE}$ with the maximal entropy based on $\theta _0$. It is impossible, however, to get an optimal hyperparameter before any observation is taken, so the resulted regression performance is therefore bound by the suboptimal hyperparameter. It should also be noted that typical patient RVs exhibit abnormal electrical patterns and that if the hyperparameter used is shared between all RVs, it implies that each RV has a similar electrical pattern, which is not the case during an arrhythmia.

An implicit exploration (IE) approach, adapted from the Thompson Sampling [12] and the implicit exploration [6] methods, addresses the discussed limitations. The kernel hyperparameter is represented by a random variable $\varvec{\theta }_{\mathbf {A}}$, and this variable has a distribution $p(\varvec{\theta _{\mathbf {A}}})$, which is updated when the set ${\mathbf {A}}$ is extended with a new pair $\left( {\tilde{\mathbf {x}}}_\mathrm{IE}, {\tilde{y}}_\mathrm{IE}\right)$. The initial $p(\theta _A)$ is a discrete uniform distribution with an expected value $\mu _{\varvec{\theta }_{\mathbf {A}}} = \theta _0$, where $\theta _0$ is from the prior knowledge, and $p(\varvec{\theta }_{\mathbf {A}})$ is updated by regularized particle filter (RPF) [8]. RPF is based on sequential importance resampling (SIR) particle filter [4] to deal with the *degeneracy* problem, which is that only a small number of the particles remain significant weights after several iterations. RPF avoids the *sample impoverishment* problem of SIR by adding a *resample-move* step in each particle generation to move each particle according to the Epanechnikov kernel. This is equivalent to resampling from a continuous approximation of the posterior [1] and has the effect of shifting the particles towards a stationary state, thereby keeping track of the target.

Compared to using a predefined kernel hyperparameter to estimate the posterior entropy in Eq. (2), IE selects the next mapping point with maximum posterior entropy based on $p(\varvec{\theta }_{\mathbf {A}})$, as shown in Eq. (3).

3 $$\begin{aligned} {\tilde{\mathbf {x}}}_\mathrm{IE} = \mathop {{{\mathrm{arg\,max}}}}\limits _{\mathbf {x'} \in \bar{{\mathbf {A}}}} {\mathbb {H}}_{p(\varvec{\theta }_{\mathbf {A}})}[\mathbf {f'} |\mathbf {x'}, {\mathbf {A}}, \varvec{\theta }_{\mathbf {A}}] \end{aligned}$$

**Fig. 2:**
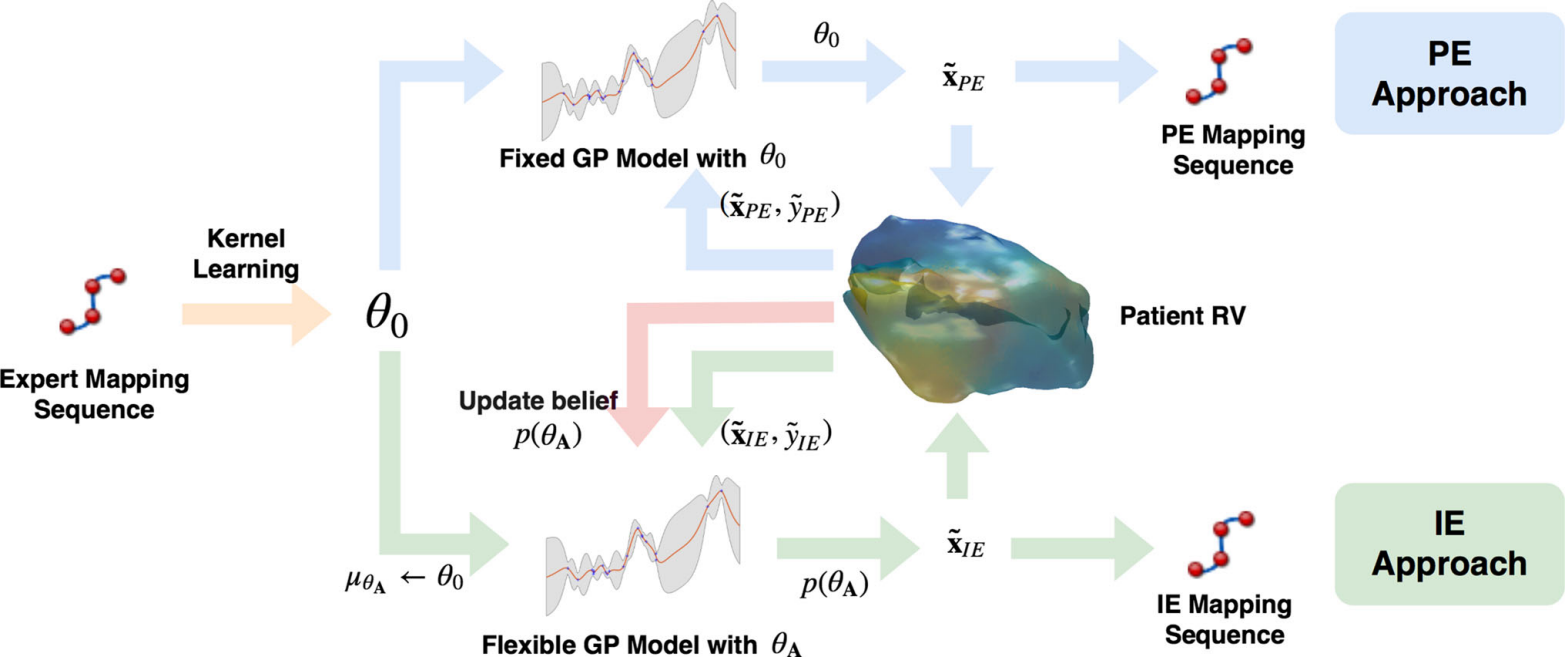
Illustration of the PE and IE approaches. While both take new observations from the RV to output new mapping points from the GP, IE constantly refines the GP kernel hyperparameter $\varvec{\theta }_{\mathbf {A}}$ with the observation

Algorithm 1 shows the complete IE algorithm when a new observation $\left( {\tilde{\mathbf {x}}}_\mathrm{IE}, {\tilde{y}}_\mathrm{IE}\right)$ is made. During the process, each new observation optimizes the surrogate model, with an ongoing exploitation when the next best mapping point is being considered. As such, IE balances the exploration–exploitation trade-off. A comparison of PE and IE is shown in Fig. 2. As the GP model is updated constantly towards the real data, it is more flexible in the face of unseen electrical map values than PE.
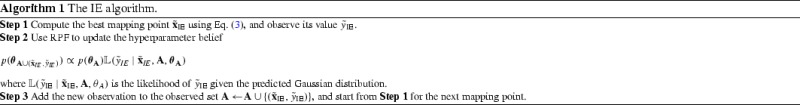

Finally, to consider the travel cost for a mapping catheter, the next mapping point ${\tilde{\mathbf {x}}}_\mathrm{IE-dist}$ is

4 $$\begin{aligned} {\tilde{\mathbf {x}}}_\mathrm{IE-dist} = \mathop {{{\mathrm{arg\,max}}}}\limits _{\mathbf {x'} \in \bar{{\mathbf {A}}}} \frac{{\mathbb {H}}_{p(\varvec{\theta }_{\mathbf {A}})}[\mathbf {f'} |\mathbf {x'}, {\mathbf {A}}, \mathbf {\theta _A}]}{{\mathbf {s}}} \end{aligned}$$

where ${\mathbf {s}}$ is the Euclidean distance between ${\mathbf {x}}'$ and the current mapping point. Also, to avoid the mapping sequence from collapsing, a minimal distance $s_\mathrm{min}$ is set so that the points within distance $s_\mathrm{min}$ are not considered for the next mapping point. The value of $s_\mathrm{min}$ is tuned to balance the number of mapping points and the size of the RV.

### Experiments

Four experiments were performed to validate the PE and IE methods and compare them with the existing geometry method given in [15]. We refer to the PE and IE methods associated with travel cost as *with travel cost* and use *without travel cost* for the case without incorporating travel cost. The geometry method [15] presented a ratio of point distance and surface curvature to be considered during path planning and thus we refer to the planning with ratio $=$ 1 as the *geometry (distance only)* method and ratio $=$ 0.5 as the *geometry (distance and curvature)* method.

Computation of mapping sequences from IE, PE and geometry methods were performed in MATLAB 2016a on a MacBook Pro with 3.1 GHz Intel Core i7 Processor. GPML toolbox version 3.6 [10] was used for kernel calculation and GP regression. For dataset 11 with 7514 vertices in the preoperative mesh, it took approximately 0.25 and 18 s to compute each mapping point for the PE and IE algorithms with 100 particles in RPF, respectively. The computation time for each mapping point was similar for all datasets.

#### 2D grid mapping simulation

A 2D grid mapping task was performed with the goal of maximizing the estimation accuracy with $m=30$ mapping points for the geometry and IE (without travel cost) methods. The ground-truth values of the query points were generated from the summation of *k* 2D Gaussian mixtures of randomly selected mean and variance and 3600 query points were evenly distributed in a $10 \times 10$ area. Four groups of experiments were conducted with $k = 5, 20, 40$, and 60 Gaussian mixtures, respectively, to represent various voltage maps from simple to complex. 20 trials were run in each experiment group. GP regression was used in both methods to estimate the 2D grid values.

**Fig. 3:**
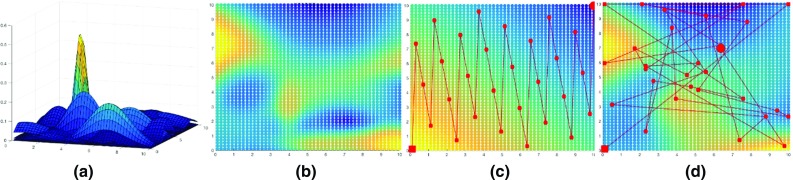
Experiment setting on an example trial with 20 random Gaussian mixtures. **a** The randomly generated Gaussian mixtures. **b** The ground-truth values generated from (**a**). **c**, **d** The mapping sequences from geometry method and the IE method and their estimated colour-coded grid values. The sequence starts from the *red square* and ending at the *large red circle*. The estimated values of (**d**) are closer to the ground-truth values in (**b**) than (**c**)

The performance of the 2D grid mapping task was measured by the structural similarity (SSIM) index [14], which evaluates the similarity of the 2D patterns of the estimated values and the ground truth. The SSIM indices of the geometry and IE methods were compared in each trial. As there was no correlation of ground-truth values between different trials, kernel learning is not possible. Thus, the prior knowledge was set as $\theta _0$ was set as $l = 1$ and $\delta _f = 1$ for the Matérn kernel. The experiment setting and the resulted mapping sequences are illustrated in Fig. 3.

#### In silico voltage mapping on retrospective patient data

**Fig. 4:**
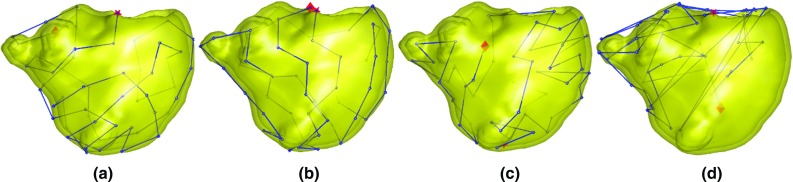
Mapping sequences (starting from the *red star* and ending at the *diamond*) on dataset 11 from the expert mapping. The *top left* of the meshes corresponds to the RV outflows, and the *top right* corresponds to the tricuspid valves. Mapping sequences: **a** IE (with distance), **b** PE (with distance), **c** Geometry (distance only) and **d** the projected expert. In (**a**) and (**b**), each mapping step was uniformly spaced with a generally consistent forward direction without explicit programming

The policies and the projected expert mapping sequences were also tested in silico in leave-one-out experiments on the 25 RV datasets from ToF patients exported from the CARTO^®^ system. Figure 4 shows the mapping sequences of dataset 11 for each algorithm. The mean L1 distance of the estimated voltage values over the vertices and the ground-truth voltage data was used to evaluate the mapping efficiency.

#### Phantom experiments with robotic catheters

Under the scenarios with a travel cost being considered, the IE and geometry (distance only) methods generated the best regression results in Table 2. Therefore, the resulted mapping sequences of these two algorithms, together with the original expert mapping sequence, were used in the robotic catheter experiments.

Dataset 11 was picked as the target RV for voltage mapping and the pre-collected voltage data in dataset 11 was used for simulating a patient RV. The voltage data were invisible to the algorithm before mapping. The mapping points can be fully computed using Algorithm 1 on the simulated voltage map. Note that only the first mapping point was needed for input and each of the subsequent mapping points can be computed iteratively based on the previous observed points.

**Fig. 5:**
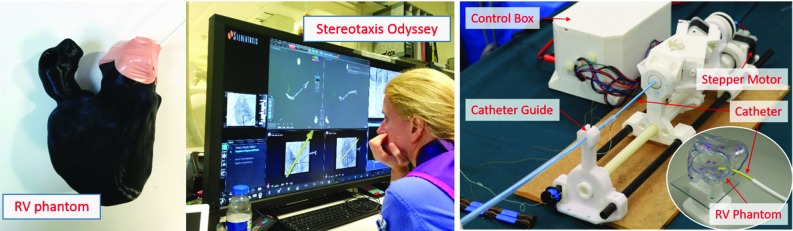
The two robotic catheter experimental set-ups: (*middle*) with the Stereotaxis Niobe^®^ and (*right*) with the new tele-operated robotic catheter. The *top left* of the RV phantom corresponds to the RV outflow, and the *top right* corresponds to the triscuspid valve

*Stereotaxis Niobe* ^®^ *robotic catheter* The Niobe^®^ remote magnetic navigation system (Stereotaxis, St. Louis, MO, USA) was used to perform voltage mapping in a phantom heart with 3D positioning provided by the CARTO^®^ 3 navigation system at the Royal Brompton Hospital, London, UK. The operator controlled the robot on the master side by manipulating the orientation of the magnetic field. The Stereotaxis Odyssey display combined all sources of data during the procedure (Fig. 5, left). The initial mapping used X-ray images, without the guidance from the proposed method, and for the second mapping, the guidance was presented on a laptop alongside the operator. The 3D cardiac phantom (RV, right atrium and pulmonary artery) was rapid prototyped in PLA and the introducer sheath was positioned at the tricuspid valve. An expert operator and a novice operator were asked to perform voltage mapping without guidance first and then with guidance for 52 mapping points.

**Table 1:** Average SSIM indices evaluating the similarity (the higher the better) between the estimated values and the ground truth

Mapping methods	5 Mixtures	20 Mixtures	40 Mixtures	60 Mixtures
Geometry	0.52	0.33	0.28	0.12
IE	0.53	0.42	0.40	0.23

There were 20 trials for each experiment group with 5, 20, 40 and 60 Gaussian mixtures, respectively. The advantage of the IE method over the geometry method becomes more apparent as the number of mixtures increases

**Table 2:** The average regression error of the expert mapping and the planning methods on the 25 datasets

Method	Regression error	
Expert mapping	0.894	
Geometry (distance only)	0.581	
Geometry (distance and curvature)	0.697	
PE	0.555 (without travel cost)	0.625 (with travel cost)
IE	0.563 (without travel cost)	0.597 (with travel cost)

The IE method produced the smallest regression error

*Tele-operated robotic catheter* A tele-operated catheter robot [3] (Fig. 5, right), which controls a manual catheter, was deployed to simulate mapping in a RV. This robot has 3 degrees of freedom, namely bending, rotation and insertion, all actuated by stepper motors, and a 3D motion controller (Wii U Nunchuk^®^, Nintendo Inc., Japan) provided input on the master side. The 3D-printed RV phantom, again from dataset 11, was rapid fabricated in resin using SLA. Mapping targets were marked on the model following the three sequences, i.e. from IE (Fig. 4a) geometry (distance only, Fig. 4c), and the projected original expert mapping sequence (Fig. 4d). The catheter tip was moved to these points by tele-operation. To reduce any human factors, the mapping experiments for the three sequences were conducted by a single operator.

**Fig. 6:**
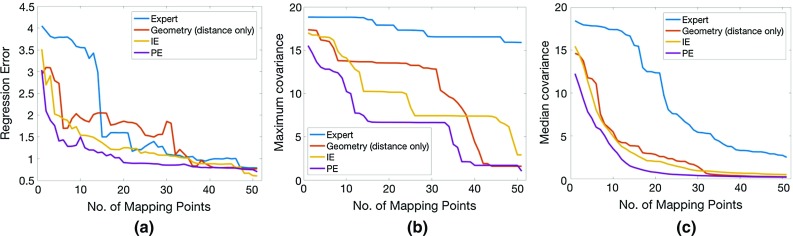
The learning and uncertainty curves of different methods on dataset 11 for the expert mapping (*blue*), geometry with distance only (*red*), PE strategy (*purple*) and IE strategy (*yellow*). **a** The mean L1 error of the estimated values on all vertices. **b**, **c** The maximum and median uncertainty over the unobserved vertices during mapping, respectively. In all, the IE and PE methods produced the fastest decrease in both the regression error and uncertainty

**Fig. 7:**
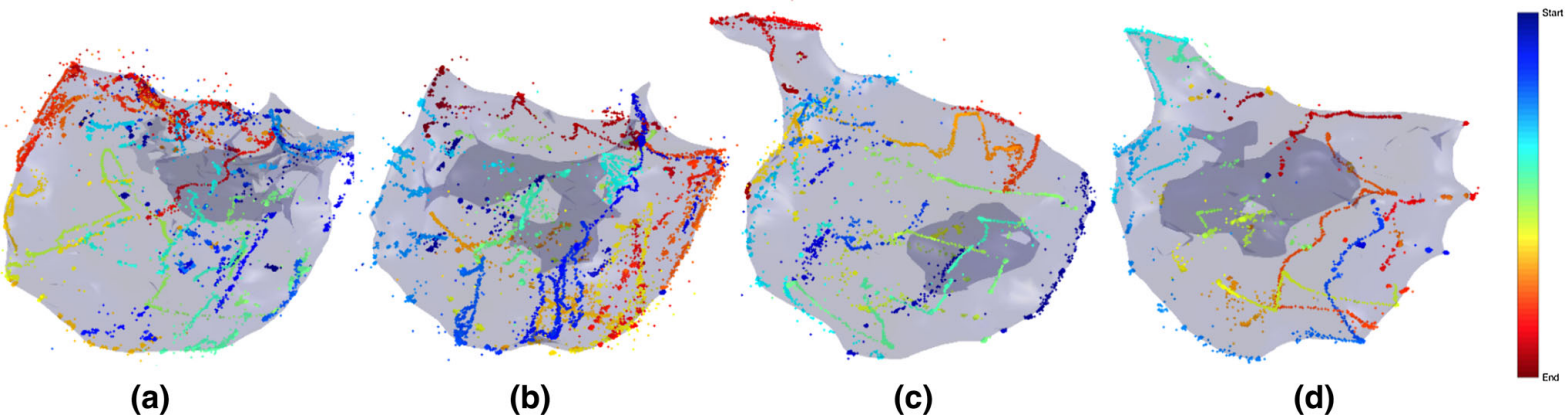
Trajectories of the mapping catheter and the resultant intraoperative meshes with the Stereotaxis catheter. The procedure time is shown from *blue* (start) to *red* (end). The *top left* corresponds to the RV outflow tract and the *top right* corresponds to triscuspid valve. **a** Novice mapping without guidance (mapping time $=$ 30 m 18 s). **b** Novice mapping with guidance (mapping time $=$ 30 m 42 s). **c** Expert mapping without guidance (mapping time $=$ 12 m 48 s). **d** Expert mapping with guidance (mapping time $=$ 11 m 26 s)

**Table 3:** Time, travel cost and operation cost of the tele-operated robotic catheter on the dataset 11

Mapping sequence	Total time (s)	Total travel distance (mm)	Total robot operations (no. steps)
IE (with distance)	579.658	1036.9038	344,030
Geometry (distance only)	579.027	1038.5505	378,841
Expert mapping	627.082	1329.7556	487,416

No significant difference was found in travel time and distance between the IE method and geometry method, but the robot operation cost was reduced by 9.18% with the IE method

## Results

Table 1 shows the SSIM index of the 2D grid mapping experiment for each method. The regression errors from the in silico voltage mapping experiments are shown in Table 2. Figure 6 shows the regression error and the maximum and median uncertainty measurement with respect to the number of mapping points taken. These measures, respectively, reveal the mapping sufficiency and the whole model uncertainty and during the procedure.

For the phantom experiments, catheter tip trajectories and operation time exported from the Niobe system for the mapping sequences were recorded. This information is shown in Fig. 7 for both novice and expert operators, with and without guidance. Finally, the operation time and costs from the tele-operated robotic catheter are presented in Table 3.

## Discussion

The simulated 2D grid data experiment (Table 1) showed the advantage of the proposed IE approach over the existing geometry approach. Across different experiment groups, this advantage became more apparent as the number of mixtures increased. In the in silico patient RV mapping experiment (Table 2), all planning methods resulted in improved mapping sequences over the expert in terms of the estimation error. The proposed IE method produced the most stable output in terms of the final regression error and the fastest decrease in both regression error and uncertainty in Fig. 6a. Although PE achieved a good estimated map without an associated travel cost, it cannot adjust the mapping decision to a different voltage data; therefore, it was suboptimal on a different trajectory that counted the travel cost. The geometry method in [15] performed favourably at an extreme case where no curvature was considered but when both the distance and the curvature were counted, its estimation was worse than IE, as more mapping points clustering at high-curvature areas revealed less voltage information about the entire chamber.

The GP posterior covariance, which reflects the model uncertainty, can also indicate mapping sufficiency. The maximal uncertainty curve in Fig. 6b exhibited three stages with IE and PE, corresponding with the expert’s practice of voltage mapping: first outlining the entire geometry, then extracting more details, and finally focusing on specific areas of interest. Figure 6c shows that the median uncertainty of IE, PE and geometry-based algorithms monotonically decreased in an S-shaped curve. By observing its slope and value, an operator can understand the mapping progress and prevent excessive mapping time and effort.

The mapping sequences of the PE and IE methods demonstrated a sequential nature that was easily integrated with any type of mapping catheter. In Fig. 4a, each mapping step was uniformly spaced with a generally consistent forward direction. This sequential behaviour was not programmed explicitly as in the geometry method but arose as a result of performing active learning for mapping.

In the phantom experiment carried out by the novice and expert operators, guidance from the IE algorithm showed benefits both subjectively and objectively. The expert operator confirmed that after taking the required mapping points for the guided procedure, no further mapping points were needed. The catheter trajectory of the expert operator with guidance (Fig. 7d) was tidier and more certain than without guidance (Fig. 7c). The novice operator’s map covered the endocardium more evenly with the guided trajectory (Fig. 7b). Without guidance, the novice operator spent more time mapping around the outflow tract and the tricuspid valve (Fig. 7a). The total mapping time taken was reduced for the expert surgeon with guidance but did not change for the novice surgeon. We believe much of this time was taken to refer to the guided points on the separate screen.

The experiment result with the tele-operated robotic catheter shows the mapping sequence from the IE method is optimal with respect to the travel time, distance travelled and robot operation cost, with a reduction of 7.56, 22.02 and 29.42%, respectively, over the original expert mapping sequence. No significant difference was found in travel time or distance between the IE method and geometry method, but the robot operation cost was reduced by 9.18% with the IE method.

## Conclusion

A new online strategy for cardiac voltage mapping by extending a GP model-based IE algorithm was proposed. The GP surrogate model which computed the mapping points learned the prior knowledge from expert mapping procedures and was simultaneously updated with RPF to fit for a specific RV. Our experiments showed that the proposed IE strategy created a better cardiac voltage map and was more time-efficient over an existing geometry-based method. The proposed mapping method provides guidance in a collaborative setting and has the potential to conduct an automated cardiac mapping process with a catheter robot.
